# Dopamine Receptor Activation Increases HIV Entry into Primary Human Macrophages

**DOI:** 10.1371/journal.pone.0108232

**Published:** 2014-09-30

**Authors:** Peter J. Gaskill, Hideaki H. Yano, Ganjam V. Kalpana, Jonathan A. Javitch, Joan W. Berman

**Affiliations:** 1 Department of Pathology, Albert Einstein College of Medicine, Bronx, New York, United States of America; 2 Department of Psychiatry and Pharmacology, Columbia University, New York, New York, United States of America; 3 Department of Genetics, Albert Einstein College of Medicine, Bronx, New York, United States of America; 4 Department of Microbiology & Immunology, Albert Einstein College of Medicine, Bronx, New York, United States of America; South Texas Veterans Health Care System and University of Texas Health Science Center at San Antonio, United States of America

## Abstract

Macrophages are the primary cell type infected with HIV in the central nervous system, and infection of these cells is a major component in the development of neuropathogenesis and HIV-associated neurocognitive disorders. Within the brains of drug abusers, macrophages are exposed to increased levels of dopamine, a neurotransmitter that mediates the addictive and reinforcing effects of drugs of abuse such as cocaine and methamphetamine. In this study we examined the effects of dopamine on HIV entry into primary human macrophages. Exposure to dopamine during infection increased the entry of R5 tropic HIV into macrophages, irrespective of the concentration of the viral inoculum. The entry pathway affected was CCR5 dependent, as antagonizing CCR5 with the small molecule inhibitor TAK779 completely blocked entry. The effect was dose-dependent and had a steep threshold, only occurring above 10^8^ M dopamine. The dopamine-mediated increase in entry required dopamine receptor activation, as it was abrogated by the pan-dopamine receptor antagonist flupenthixol, and could be mediated through both subtypes of dopamine receptors. These findings indicate that the effects of dopamine on macrophages may have a significant impact on HIV pathogenesis. They also suggest that drug-induced increases in CNS dopamine may be a common mechanism by which drugs of abuse with distinct modes of action exacerbate neuroinflammation and contribute to HIV-associated neurocognitive disorders in infected drug abusers.

## Introduction

Human Immunodeficiency Virus type 1 (HIV) enters the central nervous system (CNS) within 8 days of initial infection [1] and leads to the development of HIV-associated neurological disorders (HAND) in 40–70% of individuals [2]–[6]. Macrophages and other cells of the monocytic lineage are the primary targets for HIV infection in the CNS [7]–[10], although HIV also infects astrocytes [8], [11], [12]. Macrophages are critical to HIV mediated neuropathogenesis [13]–[16] and may serve as viral reservoirs within the CNS [17], [18]. Macrophages also release inflammatory mediators and neurotoxic viral and host proteins, contributing to chronic neuroinflammation and neurotoxicity [16], [19], [20]. Thus, infection of CNS macrophages is central to HIV-associated neuroinflammation and neurocognitive dysfunction.

Macrophages in the CNS are exposed to dopamine, a catecholamine neurotransmitter that is increased by the use of illicit drugs such as cocaine and methamphetamine [21], [22], as well as by legal therapeutics such as Ritalin and some antidepressants [23], [24]. Studies in SIV-infected rhesus macaques show that increases in extracellular dopamine correlate with increased CNS viral loads [25], [26]. HIV-infected individuals show exacerbated neuropathology in regions of the brain with high levels of dopamine, such as the basal ganglia and substantia nigra [27]–[32]. Dopamine acts principally through dopamine receptors (DR), G-protein coupled receptors (GPCR) that are divided into D1-like DR (D1R and D5R) and D2-like DR (D2R, D3R and D4R) depending upon whether they activate (D1-like DR) or inhibit (D2-like DR) adenylyl cyclase [33]. Studies show that DR also activate alternative pathways, including mobilization of calcium from the endoplasmic reticulum [34]–[37]. The effects of dopamine on macrophage function, and the signaling pathways by which these effects are mediated, have not been studied extensively.

Our previous studies showed that dopamine increases HIV replication in human macrophages through activation of DR, increasing the total number of infected cells [38]. The mechanism(s) by which this occurred are unclear, but one possibility is by increasing HIV entry into macrophages. HIV entry is complex, and in macrophages, it is mediated by the interaction of the viral envelope protein gp120 with the surface receptor CD4 and co-receptor CCR5 [39]. In this study, we examined whether dopamine increases HIV entry and whether that increase was mediated by changes in CCR5 expression and/or activation of DR.

Our data showed that dopamine increased HIV entry into human primary monocyte-derived macrophages (MDM) by approximately 2-fold, and that the increased entry occurred at dopamine concentrations above 10^−8^ M. The increased entry required CCR5, but was not mediated through changes in the surface expression of this receptor. Increased entry also required activation of DR and was mediated by both D1-like and D2-like DR, suggesting that a common DR signaling mechanism mediates the increased entry. Using transfected HEK293 cells, we demonstrated that calcium mobilization resulting from activation of G_αq_-coupled receptors, such as CCR5 [40], can be potentiated by both D1-like and D2-like DR. These data indicate that the dopamine-induced increase in macrophage HIV replication we previously reported is due, at least in part, to an increase in viral entry, and suggest that this could be a result of a dopamine-mediated increase in calcium mobilization.

## Methods

### Reagents

RPMI-1640, penicillin/streptomycin (P/S), 10X HEPES and 10X HBSS from Life Technologies (Carlsbad, CA). Human AB serum from Lonza (Basel, Switzerland). Fetal Bovine Serum from Lonza for MDM culture and from Gemini (West Sacramento, CA) for HEK293 culture. BSA, Acetylcholine, Probenecid, Dopamine, SKF81297, Sulpiride and SCH23390 from Sigma-Aldrich (St. Louis, MO). SKF38393 and Flupenthixol dihydrochloride from Tocris Biosciences (Minneapolis, MN). Quinpirole from Tocris or Sigma-Aldrich. All DR agonists and antagonists were resuspended in distilled H_2_O. TAK779 was obtained through the NIH AIDS Reagent Program, Division of AIDS, NIAID, NIH [41]. Macrophage colony stimulating factor (M-CSF) was from Peprotech (Rocky Hill, NJ), and was resuspended at 100 µM in distilled H_2_O.

### Cell isolation and culture

Human peripheral blood mononuclear cells (PBMC) were separated from blood obtained from healthy donors (New York Blood Center, Long Island City, New York) by Ficoll-Paque (GE Healthcare, Piscataway, NJ) gradient centrifugation. Monocytes present in the PBMC were determined by flow cytometry of CD14+ cells in the PBMC using a FACS Canto II flow cytometer (Becton-Dickinson, Franklin Lakes, NJ). Monocyte derived macrophages (MDM) were obtained by adherence isolation, through culture in macrophage media (RPMI-1640 with 10% FCS, 5% human AB serum, 10 mM HEPES, 1% P/S, and 10 ng/mL M-CSF) for 3 days, washing and culturing another 3–5 days. After 6–8 total days in culture the cells were considered to be mature MDM. Flp-In T-Rex HEK 293 cells (HEK 293 cells, Life Technologies) were maintained in DMEM supplemented with 10% FBS and 2 mM L-glutamine (Life).

### Generation of Viral Stocks

Viral DNA was prepared by isolating plasmids from bacterial stocks (HB101 cells, Life Technologies) transformed with viral DNA clones and purified using CsCl_2_ gradient ultracentrifugation. Stocks of HIV_BaL_ and HIV_BaL_ harboring Vpr-β-lactamase were prepared in parallel by co-transfecting 3 µg of HIV_BaL_ DNA (obtained through the NIH AIDS Reagent Program, Division of AIDS, NIAID, NIH: pWT/BAL), along with 15 µg of either pcDNA or pMM310 (a plasmid expressing Vpr-β-lactamase, obtained through the NIH AIDS Reagent Program, Division of AIDS, NIAID, NIH: pMM310 (Cat#11444) from Dr. Michael Miller [42]) into HEK 293 cells using calcium phosphate precipitation as described by the manufacturer (Chemicon). A 100% HEK 293 cells were split 1:6 in a 10 cm^2^ plate 24 hours prior to transfection, with the goal of 30–40% confluency at the time of transfection. Media was changed at 16 hours post-transfection and collected 24 and 48 hours later. Supernatant was passed through a 0.45 µm cellulose acetate syringe filter (Corning, Tewksbury, MA), and treated with 20 U/ml DNAseI (Roche, Indianapolis, IN) for 30 minutes at 37°C. Viral stocks were purified and concentrated by passing the virus through 20% sucrose/PBS gradient centrifugation for 2 hr at 36,000 rpm, 4°C, and then viral pellets were resuspended in macrophage media and stored in aliquots at 70°C. To insure Vpr-β-lactamase virions maintained infectivity, they were tested by infecting MDM and compared to infections with non-Vpr-β-lactamase containing viral stock. Analysis of p24 production in response to infection showed no significant difference in infectivity of virus with and without Vpr-β-lactamase (data not shown). To Infectious units in each viral stock were determined by infection of Hi-5 GHOST cells (obtained through the NIH AIDS Reagent Program, Division of AIDS, NIAID, NIH: GHOST Cell Transfectants - GHOST (3) Hi-5 from Dr. Vineet N. KewalRamani and Dr. Dan R. Littman [43]). Determination of the infectious units in each viral stock enabled inoculation using multiplicity of infection (MOI), insuring that all infections were performed with identical numbers of infectious virions per macrophage.

#### Viral Entry Assay

Entry assays were performed using the Geneblazer in vivo detection kit (Life Technologies) according to the manufacturers protocol, with optimization for use in primary macrophages as previously described [44]. Mature MDM cultured in flat, clear bottom 96 well black plates (ThermoFisher Scientific, Waltham, MA) were infected in quadruplicate with HIV_BaL_ containing Vpr-β-lactamase, at a multiplicity of infection (MOI) of 0.002, 0.005 or 0.01, based on the CD14+ cells in the starting PBMC population. MDM were incubated for 2.5 or 4 hours at 37°C with 5% CO_2_. Dopamine or DR agonists were added concurrently with HIV, while the pan-DR antagonist flupenthixol was added 30 minutes prior to addition of virus. All DR agonists were used at 10^8^ M, as our preliminary studies indicated that this was the concentration that induced a significant increase in entry in the greatest number of donors. After incubation, MDM were washed and incubated in the dark at RT for 6 hours in 100 µl of phenol red free macrophage media and 20 µL of 6X CCF2-AM loading solution (Loading solution ratios optimized for primary macrophages as follows; 0.04:0.36:0.25:1,935 A:B:D:C). CCF2-AM contains two fluorophores connected by a lactam ring, and generates green fluorescence by means of fluorescence resonance energy transfer (FRET) when the fluorophores are in close proximity. When the lactam ring is cleaved, the FRET interaction is disrupted and the fluorescence becomes blue. In this assay, uninfected cells fluoresce green because the lactam ring is intact and infected cells fluoresce blue because the β-lactamase enzyme contained within the infecting virus cleaves the lactam ring and separates the fluorophores [45].

After incubation at RT, 12 images of each infection condition were generated using a Zeiss IX70 inverted microscope (Zeiss) and an Olympus E-620 Live View DSLR (Olympus). Volocity (Perkin-Elmer) was used to enumerate the number of infected (blue) and uninfected (green) cells in each condition. Primary cells from different donors have varying susceptibility to HIV infection, therefore the number MDM infected with HIV was variable between experiments. There was also inter-experiment variation in the effects of different concentrations of dopamine on HIV entry, likely due to donor dependent differences in the response to dopamine. To evaluate changes in HIV entry induced by each concentration of dopamine, in each donor we compared the macrophages infected in the presence of each concentration of dopamine or agonist to the macrophages infected in control infections (those cultures infected with HIV alone). The mean difference in the number of cells infected is determined by the formula, 100x (Experimental – Control) / Control, and expressed as percent increase in macrophage infection relative to the control infection (HIV alone), which is defined as a 0% increase.

### Flow Cytometry

CD14+ cells present in isolated PBMC were determined using anti-human CD14-PE (clone M5E2, BD Biosciences, Franklin Lakes, NJ) and an isotype matched negative control, IgG_2a_-PE (BD Biosciences). PBMC were stained for 30 minutes at 4°C, washed with 1% BSA in PBS and fixed with 2% paraformaldehyde (Electron Microscopy Sciences, Hatfield, PA). Immunopositive cells were analyzed by acquisition of 10,000 events on a FACS Canto II flow cytometer and analyzed using FlowJo (Treestar, Ashland OR). To analyze CD4 or CCR5 protein expression on the macrophage surface, MDM were detached from culture dishes with TrypLE Select (10X) for 30 minutes at 37°C, followed by gentle agitation and scraping. 1–2×10^5 ^MDM were stained for CCR5 or CD4 using anti-human CCR5-APC/Cy7 and CD4-FITC, with the isotype-matched negative controls, IgG_1_-APC/Cy7 or IgG_1_-FITC (BD Biosciences). Antibodies were titrated to determine the optimal concentration for staining PBMC and MDM. After subtracting out the background fluorescence from the isotype matched control, the mean fluorescence intensity (MFI) for each antigen was determined.

### Generation of Dopamine Receptor Expressing HEK Cells

Stable cell lines were generated as previously described (Han et al. 2009). Briefly, Flp-In T-Rex 293 cells (Life Technologies) were transfected with either D1R or D2R expressing plasmids using Lipofectamine 2000 (Invitrogen) according to the manufacturer’s protocol. Transfected HEK cells were selected and maintained in media with G418 (Mediatech, Manassas, VA) or Hygromycin B (Mediatech) for expression selection. Single colonies were isolated and screened for receptor expression using FACS analysis. D2s stable line refers to cells transfected with the D2R expressing plasmid SF-D2s, while D1 stable line refers to cells transfected for inducible expression of D1R expressing plasmid, 3xHA-D1. The D1-D2s double stable line refers to HEK cells stably transfected with 3xHA-D1 and SF-D2 plasmids. Cells with inducible receptors were incubated in 1 µg/ml tetracycline-containing medium overnight prior to experiments to induce expression. Generation of these cell lines enabled maintenance of consistent receptor expression levels for calcium flux assays.

### Measurement of Calcium Flux

Calcium flux was measured using the Flipr Calcium 5 Assay kit (Molecular Devices, Sunnyvale, CA) and according to the manufacturers protocol. Briefly, cells were resuspended in HBSS buffer containing 20 mM HEPES and 2.5 mM probenecid and distributed in 40 µl volumes in 96 well plates (500,000 cells/well). Fifty µl Flipr5 dye (Molecular devices) was added to each well and plates were incubated in 37°C 5% CO_2_ for 1 hour. Plates containing 10x concentrated ligand were prepared in HBSS with 20 mM HEPES. During the calcium reading, performed on a Flexstation 3 (Molecular Devices), 10 µl ligand was injected to the well at the indicated times. Intracellular calcium levels were measured every 2 sec over the course of 220 sec. Data was analyzed by Softmax Pro 5.4 (Molecular Devices) and Prism 6.0 (Graphpad, La Jolla, CA).

### Statistics

Statistics were determined using Prism 6.0. Entry assay and flow cytometry data were analyzed for normality using a D’Agostino and Pearson omnibus normality test. Normally distributed were analyzed using a two-tailed Student’s T-test and data that were not normally distributed were analyzed with Wilcoxen Matched Pairs Signed Rank Test. P < 0.05 was considered significant.

## Results

### Dopamine Increases HIV Entry into Primary Human Macrophages

**Figure 1:**
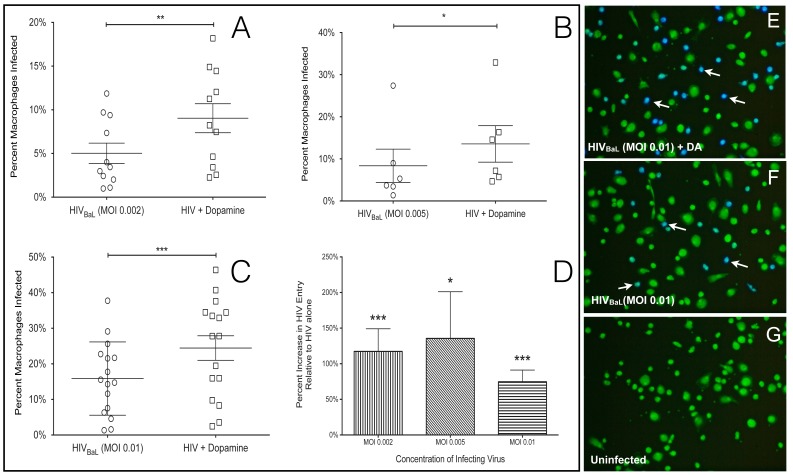
Dopamine increases HIV entry into primary human macrophages. Primary human monocyte derived macrophages were infected with 3 concentrations of β-lac HIV (MOI of 0.002, 0.005, 0.01) in the presence of either 2×10^8^ M, 2×10^7^ M, 2×10M or 2×10^5^ M dopamine, and control cells were infected with HIV alone. In panels A-C each circle or square represents infection of macrophages from a single donor. Dopamine significantly increased viral entry in macrophages infected with all concentrations of HIV (A, MOI 0.002, n = 11, ** p = 0.0002), 0.005 (B, n = 6, * p = 0.0313) or 0.01 (C, MOI 0.01, n = 16, *** p = 0.001) after 4 hr incubation. Panel D shows the percent increase in entry mediated by dopamine in infections with each concentration of HIV relative to control (MOI 0.002 vertical lines, MOI 0.005 diagonal lines, MOI 0.01, horizontal lines). Panels E - G show representative images of cultures infected with 0.01 MOI β-lac HIV in the presence of (E) dopamine, (F) HIV alone, (G) and uninfected macrophages. White arrows indicate infected cells.

To determine whether dopamine-mediated increases in HIV replication in macrophages were due to an increase in viral entry, primary human MDM were infected with different concentrations of HIV_BaL_ virions harboring an active β-lactamase enzyme (β-lac HIV). As described in the methods, entry of β-lac HIV into a macrophage causes that cell to fluoresce blue, while uninfected cells remain green. The percentage of viral entry was quantified by counting the number of blue and green cells and comparing the number of blue cells to the total cell number. Infection with β-lac HIV using a multiplicity of infection (MOI) of 0.002 for 4 hours resulted in infection of 1.0% to 11.9% of MDM, 1.3% to 27.4% using an MOI of 0.005 and 1.4% to 37.8% of cells using an MOI of 0.01 (Figure 1A, MOI 0.002, n = 11; 1B, MOI 0.005, n = 6; 1C, MOI 0.01, n = 16). The variation in the susceptibility of macrophages to infection with HIV demonstrates the heterogeneity of primary macrophages from different donors used in these studies. Treatment with dopamine significantly increased viral entry into MDM at all concentrations of HIV tested (MOI 0.002 (1A), 0.005 (1B) and 0.01(1C)). The magnitude of the dopamine-mediated increase in HIV entry resulting from infection with each concentration of HIV is shown in Figure 1D (MOI 0.002, 117% increase, n = 11, p = 0.001***; MOI 0.005, 135% increase, n = 6, p = 0.0313 *; MOI 0.01, 75% increase, n = 16, p = 0.0002 ***). These data indicate that the effect of dopamine on HIV entry is not dependent on the concentration of the infecting virus. In Figure 1D and subsequent figures showing a percent increase, the control infections (HIV alone) are defined as a 0% increase, as described in the Methods. A representative experiment showing MDM from a single donor infected with β-lac HIV at an MOI of 0.01 for 4 hours in the presence and absence of dopamine is shown (Figures 1E and 1F). Uninfected cultures showed no blue fluorescence, indicating that blue fluorescence was specifically induced by HIV entry (Figure 1G). Donor dependent differences among primary macrophages resulted in inter-experiment variation in baseline levels of HIV infection and in the magnitude of the increase in entry mediated by different concentrations of dopamine. To account for this variability, each infection was performed with 4 different concentrations of dopamine, 2×10^8^ M, 2×10^7^ M, 2×10^6^ M and 2×10^5^ M, to ensure that cells from each donor were treated with a concentration of dopamine that induced an optimal response. For each donor, the dopamine concentration that induced the maximal increase in entry was used for analysis.

**Figure 2:**
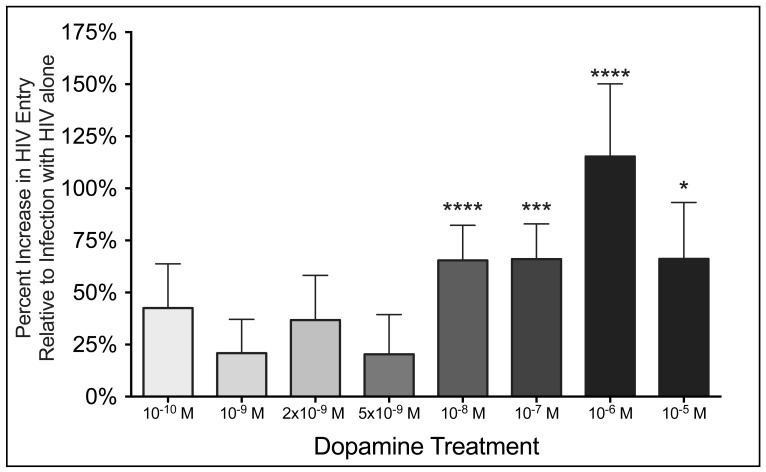
Increase in HIV entry requires a minimum threshold of dopamine. MOI 0.01 β-lac HIV was added to macrophages in the presence of 10^10^, 10^9^, 2×10^9^, 5×10^9^, 10^8^, 10^7^, 10^6^ M and 10^5^ M dopamine and control cells were infected with HIV alone. Relative to control infections, viral entry was significantly increased when MDM were infected with HIV in the presence of 10^8^, 10^7^, 10^6^ M and 10^5^ M dopamine (n = 1032, ** p < 0.01, *** p < 0.001 vs. HIV only) but not in the presence of 10^10^, 10^9^, 2×10^9^, or 5×10^9^ M dopamine. There was no significant difference in the magnitude of the increase in entry among cultures infected the presence of 10^8^, 10^7^, 10^6^ M and 10^5^ M dopamine.

To characterize further the effects of dopamine on entry, β-lac HIV (MOI 0.01) was added to MDM for 2.5 hours in the presence of 8 different concentrations of dopamine, ranging from 10^10^ M to 10^5^ M. Infection time was decreased to 2.5 hours to maximize the detection of the changes in entry, and MOI of 0.01 was used because this concentration provided the most consistent infection among donors. In experiments using cells derived from 32 different donors, addition of 0.01 MOI of β-lac HIV for 2.5 hours resulted in infection of 12.1 +/1.5% of MDM. The mean percentage of macrophages infected with HIV was significantly increased in the presence of the four highest concentrations of dopamine, 10^8^, 10^7^,10^6^ and 10^5^ M, (Figure 2, 10^8^ M DA, 72% increase, n = 32, p < 0.0001 ****; 10^7^ M DA, 73% increase, n = 21, p = 0.0005 ***; 10^6^ M DA, 115% increase, n = 15, p < 0.0001 ****; 10^5^ M DA, n = 13, p = 0.0171 *). Although a few donors showed increases in entry in response to concentrations of dopamine below 10^8^ M dopamine, the mean amount of entry in MDM treated with less than 10^8^ M dopamine was not significantly increased above the control infection with HIV alone (Figure 2, p > 0.05 for all conditions, 10^10^ M DA, n = 15; 10^9^ M DA, n = 21; 2×10^9^ M DA, n = 10; 5×10^9^ M DA, n = 10). These data show that the dopamine-mediated increase in HIV entry into macrophages is dose dependent with a steep threshold, only occurring at concentrations of 10^8^ M or greater of dopamine.

### Dopamine-Mediated Increase in HIV Entry Requires CCR5

**Figure 3:**
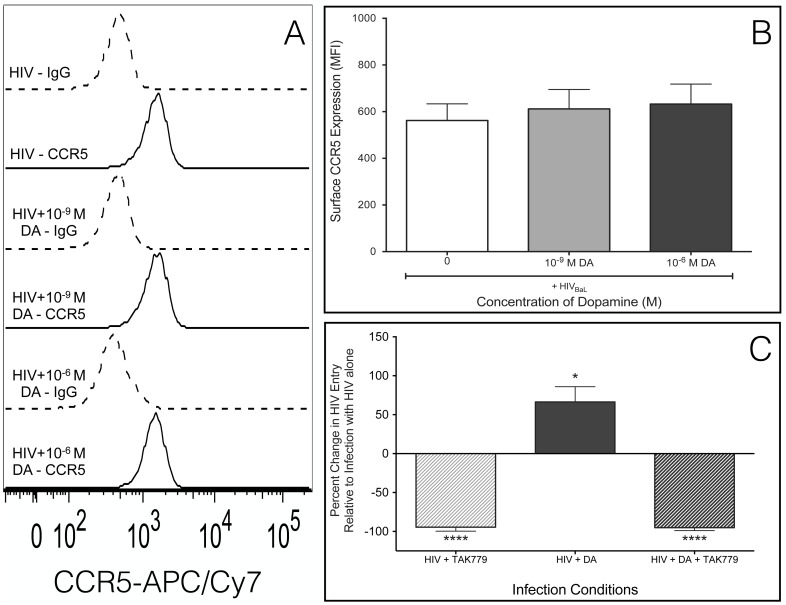
CCR5 is necessary for dopamine-mediated increase in HIV entry. Macrophages were infected with HIV_BaL_ in the presence of 10^9^ M or 10^6^ M dopamine and analyzed by flow cytometry for CCR5. (A) A representative histogram of CCR5 surface expression in a single donor in response to HIV alone (IgG – dashed grey lines, CCR5 – solid grey lines) is shown. (B) The mean MFI of CCR5 on the surface of macrophages from seven donors infected with HIV alone (white), HIV + 10^9^ M DA (light gray) or HIV + 10^6^ M DA (dark gray) relative HIV alone. Neither concentration of dopamine-induced significant changes in the expression of this protein on the cell surface relative to the macrophages infected with HIV alone. (C) Macrophages were pretreated with 2×10^7^ M TAK779, and then an MOI of 0.01 β-lac HIV was added in the presence or absence of 10^8^ M dopamine. As controls, both macrophages pretreated with TAK779 and macrophages not pretreated were infected with HIV alone. Dopamine significantly increased HIV entry (n = 6, p = 0.0194 *), and pretreatment with 2×10^7^ M of the CCR5 inhibitor TAK779 blocked HIV entry into macrophages infected in both the presence and absence of dopamine (HIV + TAK779, p = 0.0001 **, HIV + TAK779 + DA, p = 0.0003 ***).

In macrophages, HIV entry is generally mediated by binding of gp120 to CD4 and CCR5 on the plasma membrane, which initiates fusion of the viral membrane with the cell membrane [39]. Expression of CCR5 correlates with macrophage susceptibility to HIV [46]–[48] and increased surface CCR5 can mediate increased HIV infection [49]–[51]. Therefore, dopamine-treated macrophages were evaluated for changes in surface CCR5 by flow cytometry. MDM were treated with dopamine for 2.5 hours in the presence or absence of HIV infection, and MDM not treated with dopamine were used as controls. Dopamine concentrations of 10^6^ M and 10^9^ M were selected as representative concentrations that did or did not increase HIV entry, respectively. Neither concentration of dopamine increased surface CCR5 in HIV-infected MDM (Figures 3A, 3B, n = 7) or in uninfected MDM, and HIV infection alone did not increase CCR5 expression (data not shown). These data indicate that dopamine does not increase HIV entry into macrophages by increasing surface CCR5. Macrophages were also examined by flow cytometry for dopamine-mediated changes in surface CD4. However, CD4 receptor expression on MDM was low and varied among donors, and we were not able to quantify reliably these data (data not shown).

In the CNS, where macrophages could be exposed to high concentrations of dopamine, R5-tropic viruses predominate [52]–[54]. However, in addition to CCR5-mediated entry, HIV can enter macrophages through alternative pathways such as the endocytic pathway or through interaction with the co-receptor CXCR4 or minor co-receptors including CCR3 [53], [55]–[60]. To determine whether dopamine increases viral entry by enabling HIV to use a CCR5-independent entry mechanism, we examined dopamine-mediated changes in entry in the presence of a CCR5 inhibitor. Macrophages were pretreated with the CCR5 inhibitor TAK779 (2 ×10^7^ M) for 1 hour, then TAK779 treated and untreated MDM were infected with 0.01 MOI of β-lac HIV in the presence or absence of 10^8^ M dopamine. Dopamine treatment significantly increased HIV entry relative to cells infected with HIV alone (Figure 3C, n = 6,10^8^ M DA, 66.4% increase, p = 0.0194 *). Blocking CCR5 with TAK779 abrogated HIV entry in pretreated cultures infected with both HIV alone and with HIV + dopamine (Figure 3C, n = 6, HIV + TAK779, 94.5% decrease, p = 0.0001 **, HIV + TAK779 + DA, 95.5% decrease, p = 0.0003 ***). These data demonstrate that the dopamine-mediated increase in entry requires CCR5 and suggest that dopamine does not act through a CCR5-independent entry pathway.

### Increase in HIV Entry is Specifically Mediated by Activation of Dopamine Receptors

Studies in rodents show that dopamine can activate other types of receptors in addition to DR [61], [62]. To determine whether the dopamine-induced increase in HIV entry is specifically mediated by activation of DR, we examined entry in the presence of the pan-DR antagonist flupenthixol. This molecule binds to all DR with high affinity (K_i_ < 8×10^8^ M for all DR, [63]–[65]), and should interfere with the binding of dopamine to these receptors.

**Figure 4:**
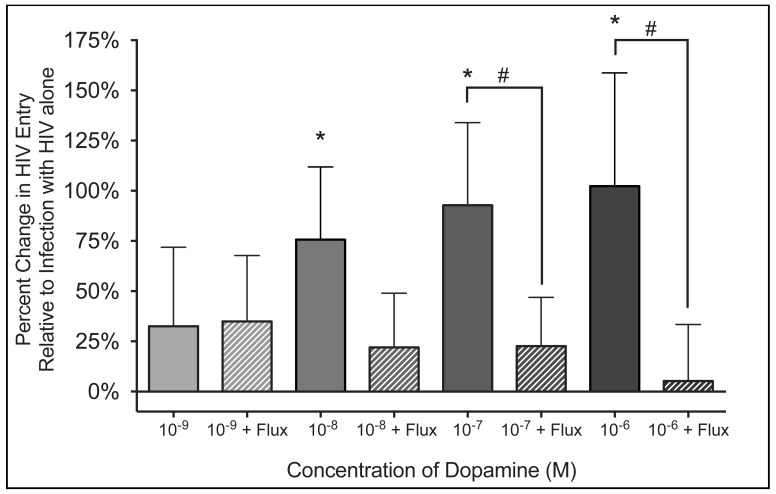
Activation of dopamine receptors required for the dopamine-mediated increase in HIV entry. Macrophages were pretreated with the pan-dopamine receptor (DR) antagonist flupenthixol (flux, 10^6^ M) and then MOI 0.01 β-lac HIV was added concurrently with either 10^9^, 10^8^, 10^7^ and 10^6^ M dopamine. As controls, both vehicle treated and flupenthixol treated cells were infected with HIV alone. Solid columns represent infections in the presence of dopamine, while hatched columns to the right of each solid column of the same color represent infections in presence of an identical concentration of dopamine as well as flupenthixol. There was a significant increase in viral entry into macrophages infected with HIV the presence of 10^8^, 10^7^ and 10^6^ M dopamine (n = 7 for all concentrations of dopamine, * p < 0.05 vs. HIV only). This increase was blocked by flupenthixol treatment, and entry was significantly reduced in the presence of 10^7^ and 10^6^ M dopamine (n = 7, # p < 0.05 vs. identical concentration of dopamine without flupenthixol).

Macrophages were infected with of β-lac HIV (0.01 MOI) and concurrently treated with 10^9^ M, 10^8^ M, 10^7 ^M and 10^6^ M dopamine. Infections were performed in presence or absence of 10^6^ M flupenthixol, added 30 minutes prior to the addition of β-lac HIV and dopamine. The presence of 10^8^ M, 10^7^ M and 10^6^ M dopamine significantly increased viral entry (Figure 4, n = 7 for all concentrations of dopamine, 10^8^ M DA 76% increase, p = 0.0156 *; 10^7^ M DA 93% increase, p = 0.0469; 10^6^ M DA, 102% increase, p = 0.0156 *). Pretreatment with flupenthixol abrogated this effect, significantly reducing HIV entry in the presence of 10^7^ M (p = 0.0313 *) and 10^6^ M dopamine (p = 0.0313 *). Fluxpenthixol did not significantly decrease the effect of 10^8^ M dopamine, although infections in the presence of 10^8^ M dopamine showed a large decrease in entry that trended toward significance (p = 0.0781). Flupenthixol treatment did not affect viral entry in MDM not treated with dopamine. These data demonstrate that activation of DR is necessary for the dopamine-mediated increase in HIV entry.

**Figure 5:**
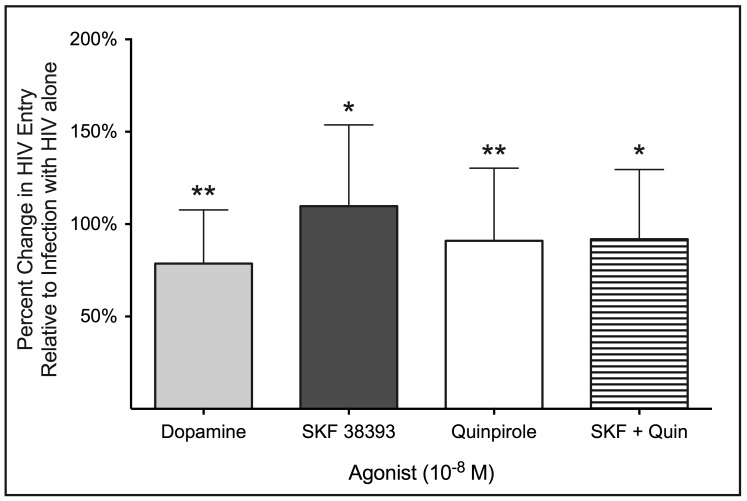
Activation of both D1-like and D2-like dopamine receptors is capable of mediating increased HIV entry. Macrophages were infected with β-lac HIV (MOI 0.01) in the presence of either 10^8^ M dopamine (light gray), a D1-like DR agonist (10^8^ M SKF 38393, dark gray), a D2-like DR agonist (10^8^ M Quinpirole, white), both SKF 38393 and Quinpirole together (horizontal lines). Macrophages infected with HIV alone served as controls. Dopamine, SKF 38393, Quinpirole and SKF 383893 + Quinpirole all significantly increased viral entry relative to infections with HIV alone (n = 10, * p > 0.05, ** p > 0.01 vs. HIV alone).

Our previous data showed that activation of D2-like DR increased viral replication [38], and more recent experiments demonstrated that D1-like DR activation did so as well (P.J. Gaskill and J.W. Berman, unpublished data). To determine the subtypes of DR that mediate the effects of dopamine on HIV entry, MDM were infected with 0.01 MOI of β-lac HIV in the presence of either dopamine, SKF 38393 (D1-like DR agonist), Quinpirole (D2-like DR agonist) or both SKF and Quinpirole together. Dopamine and all agonists were added at a concentration of 10^8^ M. Dopamine, SKF 38393 and Quinpirole each significantly increased HIV entry into macrophages (Figure 5, n = 10 for all agonists, Dopamine, 79% increase, p = 0.0039 **; SKF 38393 110% increase, p = 0.0342 *; Quinpirole, 91% increase, p = 0.0098 **). Infection in the presence of agonists for both D1-like and D2-like DR together also significantly increased entry, but did not enhance the increase in entry beyond that seen for each agonist individually (Figure 5, n = 10, SKF 38393 + Quin, 92% increase, p = 0.0375 *). The magnitude of the increase in entry did not differ significantly among any of these DR ligands. These experiments demonstrate that the activation of either D1-like or D2-like receptors mediates increased HIV entry into macrophages. The lack of an additive or synergistic effect could indicate that the two types of DR act through a common signaling pathway to increase HIV entry.

### Activation of Both D1-like and D2-like Dopamine Receptors Potentiates Calcium Mobilization Triggered by Prior Activation of G_αq_-Coupled GPCR

That HIV entry is increased by both D1-like and D2-like DR suggests the possibility that they do so through a common signaling pathway. Although D1-like and D2-like receptors have opposite effects on cAMP production, they have both been reported to cause calcium mobilization. Calcium is important for HIV infection; it is induced by binding of gp120 to CCR5 on macrophages [66], [67], and calcium mobilization mediated by activation of the inositol triphosphate receptor (IP3R) in the endoplasmic reticulum is required for HIV entry [68]. Both subtypes of DR have been reported to mediate calcium release from the endoplasmic reticulum through a potentiation mechanism, D1-like DR through activation of protein kinase A [69], and D2-like DR through interaction with phospholipase C (PLC) and calcyon [70]. In this process, activation of DR evokes an increase in Ca^2+^ mobilization following an initial Ca^2+^ release mediated by activation of a G_αq_-coupled receptor, such as CCR5. In addition, in cells heterologously expressing both D1R and D2R, coactivation of both receptor subtypes can initiate calcium release through G_βγ_-mediated signaling [35]. Thus, a common pathway through which both D1-like and D2-like DR could mediate calcium flux is G_βγ_ activation of PLCβ, as activation of both subtypes of DR release G_βγ_ subunits. This would enable activation of both subtypes of DR to increase HIV entry by potentiating the Ca^2+^ mobilization required for the entry process. To determine whether both D1-like and D2-like DR could increase calcium mobilization through potentiation of G_αo_ signaling, we examined calcium flux in HEK293 cells stably transfected with D1R, D2R, or both dopamine receptors.

**Figure 6:**
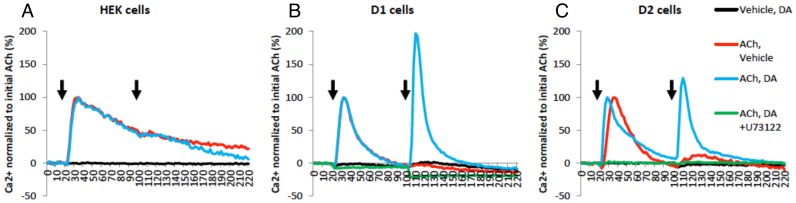
Dopamine receptors potentiate G_αq_-receptor mediated calcium flux through phospholipase C. Intracellular calcium levels were measured every 2 seconds for 220 seconds and plotted against time. Ca^2+^ level is shown in percentage normalized to the initial 10^5^ M acetylcholine (ACh) added at 20 seconds. Vehicle (red curve), 10^5^ M dopamine (DA, blue curve), or 10^5^ M DA with 10^5^ M U73122 (PLC inhibitor, green curve) was added at 100 seconds following 10^5^ M ACh at 20 secs, indicated by arrows. As a negative control, vehicle followed by 10^5^ M DA was also measured (black curve). Experiments were performed in parental (A) HEK cells, (B) D1R expressing cells, and (C) D2R expressing cells. Traces are representatives of n = 3 experiments.

To determine whether DR activation in the absence of G_αo_ activation induced calcium flux, parental HEK cells or cells transfected with either D1R or D2R were treated with 10^5^ M dopamine (Figure 6, black curves). These experiments showed that dopamine alone did not induce any calcium release. The G_αq_-mediated calcium response was tested using 10^5^ M acetylcholine (ACh) to stimulate endogenous G_αq_-coupled muscarinic receptors (M3) expressed in HEK cells [71] (Figure 6, red curves). Treatment with acetylcholine resulted in a clear pattern of prolonged (> 60 secs to subside) internal calcium release, with kinetics consistent with IP3R-mediated calcium mobilization [72]. This ACh-induced calcium flux was specific to muscarinic receptor activation as it was blocked by the muscarinic receptor-specific antagonist pirenzepine (data not shown).

**Figure 7:**
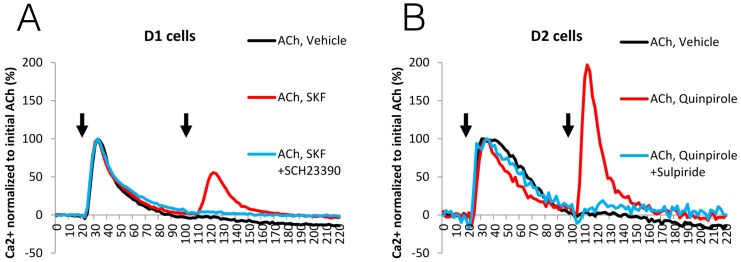
Potentiation of G_αq_-mediated calcium flux is induced specifically by activation of D1-like and D2-like dopamine receptors. Intracellular calcium levels were measured every 2 seconds and plotted against time. Ca^2+^ level is shown in percentage normalized to the initial 10^5^ M acetylcholine (ACh) added at 20 secs. (A) In D1 stable cells, either vehicle control (black curve), 10^5^ M SKF81297 (D1 agonist, red curve), or 10^5^ M SKF81297 with 10^5^ M SCH23390 (D1 antagonist, blue curve) was added at 100 secs following 10^5^ M ACh at 20 secs, indicated by arrows. (B) In D2 stable cells, either vehicle control (black curve), 10^5^ M Quinpirole (D2 agonist, red curve), or 10^5^ M Quinpirole with 10^5^ M Sulpiride (D2 antagonist, blue curve) was added at 100 secs following 10^5^ M ACh at 20 secs, indicated by arrows. Traces are representatives of n = 3 experiments.

Once G_αq_-mediated calcium release was defined, we determined whether 10^5^ M dopamine, which was inactive alone, could potentiate the effect of 10^5^ M ACh if added 80 seconds later. Treatment with dopamine following G_αq_ stimulation induced a second calcium response in cell lines expressing either D1R or D2R, but not in the control parental HEK cells (Figure 6, blue curves). In all of the cell lines, pretreatment with 10^5^ M U73122, a potent inhibitor of PLC [73], abrogated the ACh-mediated calcium mobilization, as well as the subsequent dopamine-mediated potentiation effect. (Figure 6, green curves). To validate the involvement of D1R and D2R in calcium release, selective agonists as well as antagonists were used. Using HEK cells expressing either D1R or D2R, the potentiation of G_αq_-mediated calcium release was triggered and blocked by their cognate agonists and antagonists (10^5^ M SKF81297 and SCH23390 for D1R and 10^5^ M Quinpirole and Sulpiride for D2R) respectively (Figure 7A, B). These results demonstrate the existence of specific D1-like and D2-like DR-dependent mechanisms for potentiation of G_αq_-mediated calcium mobilization through PLC activation.

## Discussion

Increases in CNS dopamine and damage to the dopaminergic system correlate with HIV-associated neuropathogenesis and HAND [31], [32], but the mechanisms underlying these correlations are not well understood. Prior to the use of combinatorial anti-retroviral therapy (cART), increased amounts of HIV DNA [74] and increased neuropathology were found in regions of the brain innervated by dopaminergic neurons, especially the basal ganglia [27], [28], [30], [75]. In SIV-infected macaques, treatment with Selegiline, L-DOPA or methamphetamine, all of which elevate CNS dopamine, increased viral replication in the CNS and exacerbated neuropathology [25], [26], [76]. These data indicate strong connection between the dopaminergic system and the development of neuropathology and HAND. Our laboratory showed that dopamine increases HIV infection in macrophages, and alters macrophage production of inflammatory cytokines [38], [77]. Although astrocytes can be infected with HIV [8], [11], [12], macrophages and other cells of the monocytic lineage are the primary target cells for the virus in the CNS [7]–[10]. This indicates that the impact of dopamine on HAND may be mediated, at least in part, by its effects on macrophage infection [78]. Thus, defining the mechanism by which dopamine increases HIV infection of these cells is important to understanding the effects of drug abuse on the development HAND.

This study demonstrates that dopamine significantly increases the entry of HIV into primary human macrophages across a range of concentrations of the infecting virus. This effect showed a very steep concentration dependence such that at and above 10^8^ M dopamine the increase in entry was of similar magnitude. The mechanism(s) underlying this steep dose dependence is unclear. The concentration of dopamine that CNS macrophages may be exposed to in the human brain is difficult to determine, but based on studies in animal models, basal dopamine levels in the CNS are estimated to be in the low nanomolar range, although some studies suggest they may be higher, particularly during active neurotransmission [79]–[82]. Dopamine concentrations also vary regionally within the CNS [83], [84], and the amount of dopamine to which macrophages could be exposed depends on the area of the brain in which they reside and the type of stimulation initiating the dopamine release [85]–[87].

The use of illicit drugs, alcohol, and certain therapeutic agents significantly increases dopamine [88]–[95]. The greatest elevation of dopamine concentrations occur within the basal ganglia, specifically in the striatum and nucleus accumbens, as well as in the ventral tegmental area and substantia nigra, which contain the dopaminergic cell bodies [87], [96]. Dopaminergic communication is mediated by volume transmission, causing dopamine to suffuse the tissue and extracellular space surrounding the releasing neurons [97]–[99]. Depending on the region, neurotransmission can evoke dopamine concentrations above 10^8^ M at substantial distance from the synapse at which the dopamine was released. Using animal models, this distance is estimated to be 7–20 µm in the striatum and 6–10 µm in the nucleus accumbens [80], [97], [99]–[102]. Thus, CNS macrophages in these regions could encounter dopamine as it effluxes from the synapse or when it is released extrasynaptically [80], [85], [87], [103], [104]. Use of drugs that increase CNS dopamine concentrations significantly expand the radius of dopamine diffusion [85]. Thus, in the CNS of drug abusers, the increased volume of tissue containing higher concentrations of dopamine would expose greater numbers of macrophages to dopamine as it diffuses into a larger area surrounding dopaminergic neurons affected by drugs of abuse.

The concentration of dopamine released in response to different drugs depends on the mechanism of action of the drug [94], [105], the age of the user [106], and the nature of the drug use, as there are large differences in dopamine response between chronic drug abusers and intermittent or naive users [107]–[109]. Some studies show that chronic drug use decreases drug-induced dopamine release [91], [107], [110], [111], suggesting that macrophages in the CNS of chronic drug users are less likely to be exposed to dopamine levels high enough to increase viral entry. However, acute and intermittent drug use could expose CNS macrophages to dopamine concentrations greater than the threshold at which dopamine increases HIV entry. Overall, these data suggest elevation of CNS dopamine may be a common mechanism by which different types of drugs increase HIV infection of macrophages and thereby contribute to neuropathogenesis and the development of HAND.

HIV infection is initiated by the entry of the virus into the target cell. In macrophages, viral entry is mediated by the interaction of the HIV envelope protein, gp120, with CD4 and a chemokine receptor, generally CCR5 [39]. The majority of viruses in the CNS, where macrophages would encounter the higher concentrations of dopamine induced by drug abuse, use CCR5 [53], [54], [112], [113], although it has been suggested that X4 viruses may infect macrophages in the later stages of HIV neuropathogenesis [52]. Increases in surface CCR5 can increase HIV infection [49], [50], [114], but neither 10^9^ M nor 10^6^ M dopamine significantly altered surface expression of CCR5. These results indicate that dopamine does not mediate its effects on entry by increasing the expression of CCR5, and suggest the possibility that dopamine may increase entry into macrophages through a CCR5-independent entry pathway, such as use of alternate co-receptors or endocytosis [55]–[58]. However, treatment with the CCR5 inhibitor TAK779 abrogated viral entry in both the presence and absence of dopamine.

These results indicate that the increase in entry is not mediated through a CCR5-independent entry pathway, as increased entry by means of this alternative pathway would not have been blocked by inhibition of CCR5. Thus, dopamine may induce other changes that enhance the entry process. Macrophage-tropic HIV from the CNS can exhibit conformational changes in gp120 that enable these viruses to infect more efficiently cells with low surface CD4 [53], [113], [115], such as macrophages and microglia [116], [117]. Increases in surface CD4 also increase viral replication [49], [114]. Therefore, a dopamine-mediated increase in this receptor could increase HIV entry. We examined CD4 expression on MDM and found that surface CD4 was low and varied in different donors. While we were unable to quantify these data reliably, they did suggest that dopamine does not increase surface CD4.

Another possibility is that dopamine-mediated changes in CCR5 could increase viral entry. CCR5 is present on the cell surface in multiple conformational states [118], and studies show different conformations of this receptor increase the binding affinity or accessibility of CCR5 to HIV, changing the efficiency of entry or fusion [119]–[121]. In macrophage-tropic CNS viruses, changes in the interaction of gp120 with the 1^st^ and 2^nd^ extracellular loop regions and the N-Terminus increased the efficiency of HIV infection [120]. Thus, dopamine-mediated changes in the expression or abundance of specific conformations of CCR5 on the macrophage surface could be responsible for the increase in HIV entry. Our study used a single virus strain derived from the lung, HIV_BaL_, so it is unclear whether dopamine-mediated changes in the conformation CCR5 could affect the entry of other strains of HIV. However, we found previously that dopamine increases the replication of HIV_ADA_ and HIV_YU2_ [38], viruses that were derived from the blood and brain, respectively [122], [123], suggesting that dopamine-mediated changes in CCR5 could increase HIV entry of R5 viruses derived from many different compartments.

In addition to CCR5, the dopamine-mediated increase in HIV entry requires DR activation and is mediated by D1-like and D2-like DR, suggesting a signaling pathway common to both subtypes of DR. The major pathway activated by DR is the modulation of cAMP, D1-like DR activating adenylyl cyclase through G_αs/olf_, and D2-like DR inhibiting it through G_αi/o_ [33], [34]. However, D1-like and D2-like dopamine receptors have opposing effects on the regulation of cAMP activity, making it less likely that this pathway is mediating the change in HIV entry. One signaling mechanism common to both D1-like and D2-like dopamine receptors is calcium mobilization, although it is unclear if calcium flux occurs through monomeric DR or heteromeric D1R–D2R complexes [35], [124]. The interaction of gp120 with CCR5 induces calcium mobilization [66], [67], [125], [126], and CCR5 activation is mediated by G_αq_ [40]. In the U87 astrocyte cell line, calcium release induced by G_αq_-mediated activation of PLC was required for successful infection [68], suggesting that a dopamine-mediated increase in calcium may increase HIV entry.

The primary pathway by which DR mediate increased calcium release from the endoplasmic reticulum is a “potentiation” mechanism. In this mechanism, stimulation of a G_αq_-coupled receptor, such as CCR5, initiates calcium release and then calcium flux is potentiated through a second pathway. Studies show that D1R-induced G_αs_- and PKA-mediated activation of IP3R [69] or D2R-induced PLC- and calcyon-mediated activation of IP3R [70] can potentiate G_αq_-mediated calcium release. Thus, it is possible that both D1-like and D2-like DR increase entry through calcium release mediated by distinct pathways, as both of these mechanisms were shown to be specific to one DR subtype. However, the data show no additive or synergistic effect on entry resulting from activation of both D1-like and D2-like DR, and if the two types of DR were acting on distinct signaling pathways, the effects on entry might be more than for either pathway alone as both pathways acted to increase entry. This suggests that the DR induced effects on HIV entry may be mediated by a common signaling pathway.

One possible common mechanism for mediating calcium release through activation of both D1-like and D2-like DR is G_βγ_ subunit-mediated PLCβ activation [127], which could be initiated by both subtypes of DR. Using our transfected HEK293 cells, we demonstrated that both D1R and D2R potentiate calcium mobilization initiated by G_αq_ activation, and that these effects were specifically mediated by activation of PLC. Thus, activation of either D1-like or D2-like DR could potentiate the G_αq_-mediated calcium flux initiated by gp120-CCR5 binding, and the resulting increase in calcium could increase HIV entry. Although this mechanism addresses the involvement of both dopamine receptor subtypes and CCR5 in the increase in HIV entry, DR signaling in primary human macrophages is poorly defined and entry could also be increased by a separate effect of DR activation. For example, DR have been reported to form oligomers with other GPCRs that alter receptor pharmacology, trafficking and signaling [128]–[132]. If DR-CCR5 heteromers were formed, this could alter the conformation or trafficking of CCR5 to enhance its ability to bind viral particles or increase the efficiency of the membrane fusion process. Thus, there are several possible mechanisms by which these receptors could mediate the increase in HIV entry. Regardless of these possibilities, our data demonstrate that dopamine alters a stage of the replication cycle between viral attachment and uncoating, the beginning and end of the entry process, respectively [133]. Studies are ongoing examining dopaminergic changes in the expression and function of host and viral proteins involved in the entry process.

Dopamine-mediated increases in HIV infection of macrophages could have a substantial impact on early HIV infection in the CNS, where most viruses are R5-tropic [112]. During acute infection, before most individuals know they are infected and/or begin therapy, infection of a greater number of CNS macrophages could increase the amount of HIV in the CNS, accelerating both the spread of infection and the developing neuroinflammation present early after CNS invasion [134], [135]. This possibility is supported by a recent study showing that methamphetamine is associated with increased neurocognitive impairment in early stage HIV infection [136]. A high percentage of HIV-positive individuals are drug users [137]–[140] and often have poor adherence to medication [141]–[145]. Therefore, an increase in viral entry into macrophages could also be significant during the course of infection with each subsequent use of a drug during a medication hiatus.

The adverse effects of dopamine on HIV replication are not limited to HIV-infected drug abusers but could also impact individuals prescribed legal therapeutics that alter dopamine levels. These drugs include L-DOPA or direct acting DR agonists for Parkinson’s disease [146], Ritalin [24], and antidepressants and psychiatric drugs such as Bupropion, a norepinephrine-dopamine reuptake inhibitor [23] or Abilify, a partial D2-like DR agonist [147]. There is a high prevalence of psychiatric disorders among HIV infected people [148]–[152]. These individuals are more likely to be prescribed drugs that modulate the dopaminergic system and thereby might increase the progression of HIV infection in the CNS.

In addition to drug-induced increases in dopamine, numerous studies have found that HIV infection by itself can alter the CNS dopaminergic system. Some studies show dopamine and dopamine metabolites are reduced in postmortem brains from HIV infected individuals [153]–[155] and in the CSF of HIV+ individuals with late stage disease [156]–[159]. However, another study showed increased CNS dopamine in the CSF of therapy-naive, HIV-infected people in early stage disease [160], [161]. Postmortem data from individuals with HIV encephalitis also suggested increased dopaminergic tone in the striatum [162]. Additionally, some studies show CNS hypermetabolism in subcortical regions such as the basal ganglia in earlier stages of infection, and subcortical hypometabolism in later stage disease [163]–[166]. Thus, HIV infection may have different effects on CNS metabolism at different stages of disease, and may potentially increase CNS dopamine in subcortical structures in early stage disease before reducing CNS dopamine during more advanced infection. A study examining changes in cerebral metabolism in drug abusers showed that intravenous drug use may have a synergistic effect with HIV on cerebral metabolism, increasing subcortical hypermetabolism and inducing the premature emergence of cortical hypometabolism relative to HIV infected non-drug users [166]. Thus, use of drugs of abuse or other substances that increase CNS dopamine during early stage infection could increase the already elevated levels of dopamine that may occur in early HIV infection of the CNS, increasing the number of macrophages exposed to higher levels of dopamine. Conversely, in late stage disease, HIV+ individuals may be less susceptible to a dopamine-mediated increase in macrophage infection due to the lower dopaminergic tone in the CNS.

The data in this study show that the number of macrophages infected with HIV is greater in the presence of increased dopamine. Elevated dopamine may significantly impact the onset and initial progression of HIV CNS infection by enabling the virus to enter more macrophages early in infection. This would increase the amount of virus in the CNS, contributing to the development of inflammation and the formation of viral reservoirs. Thus, increases in CNS dopamine may be a common mechanism by which different drugs of abuse modulate the development of HAND. Overall, these data contribute to our understanding of HIV infection of the CNS and the development of HAND in HIV-infected drug abusers and may help to guide the use of therapeutic interventions in HIV-infected drug abusers.
